# Supplementary material for: Are carbon emissions trading and green financial instruments synergistic? -Comprehensive quantitative research based on content analysis
Source: PLoS One. 2024 Mar 7;19(3):e0298601. doi: 10.1371/journal.pone.0298601 (PMC10919601; doi:10.1371/journal.pone.0298601)
Supplement: S1 Data — (ZIP) [file pone.0298601.s001.zip › Data/地级市Z_F工作报告.zip!/╣π╢1⁄2/╔╟═╖╩╨/└┤╫╘╣1⁄2╓┌║┼í╛┬φ┐╦╩2╛▌═°í┐_2004.docx]

各位代表：

我代表市人民政府向大会作政府工作报告，请予审议，并请各位政协委员和其他列席人员提出意见。2003年工作回顾

2003年，是我市经济社会在新的起跑线上健康发展的一年。一年来，在市委的正确领导下，在市人大及其常委会和市政协的监督支持下，市政府认真贯彻落实中共十六大、省第九次党代会和市第八次党代会精神，以胡锦涛总书记视察广东、张德江书记视察汕头的重要讲话精神为动力，团结带领全市人民，坚持一手抓防治非典不放松，一手抓经济建设不动摇，迎难而上，奋力拼搏，较好地完成了市十一届人大一次会议确定的年度任务，全市国民经济和社会发展取得新的成绩。———国民经济发展呈现新态势

全年实现国内生产总值526．2亿元，人均国内生产总值10856元，分别比上年增长8．8％和5．6％。财政收入45．7亿元，增长8％；其中地方一般预算收入20．8亿元，增长6．1％。信贷、工业投资、工业用电量等增长迅速，工业生产、外贸出口、投资成为拉动经济增长的主要力量。经济结构继续调整优化，经济运行质量进一步提高。

工业经济加快发展。推进“工业强市”战略，落实各种扶持工业的政策措施。全年实现工业总产值925．1亿元，增长10．1％。工业对国内生产总值增长的贡献率达到71．1％。工业经济效益指数122．6％，同比提高5．4个百分点。整合园区资源，重点园区建设进展顺利。传统产业改造升级力度加大，新增工业技改项目80项，4个重点国债技术改造项目进展顺利。实施名牌带动战略，新增省著名商标15件、省名牌产品11个、国家免检产品2个，宣传推介第三批名牌产品22个。优势行业主导作用增强，电子信息、纺织服装、工艺玩具、化工塑料、食品医药、机械装备、印刷包装、音像材料等优势产业发展壮大。澄海区被授予“中国工艺毛衫名城”称号，潮阳区谷饶镇被授予“中国针织内衣名镇”称号。

农业和农村经济进一步发展。实现农业总产值74．3亿元，增长2％。农业产业化步伐加快，种植业结构优化，优质玉米、蔬菜、水果、花卉等生产有新发展。汕头农业现代化示范区被列为省级示范区。农业标准化工作取得显著成效，制订发布农业地方标准33项，设立国家级农业标准化示范区1个。农产品质量安全体系建设加快，省级无公害农产品生产基地和水产品产地认证39个。加强海域使用管理，推进渔业结构调整，海水养殖产量增长20％，全市水产品出口量居全省第一。南澳成为全国最大的县级太平洋牡蛎养殖基地。林业生态环境建设取得新进展。防灾减灾工作成效明显。农业基础设施建设得到加强，共投入农业综合开发土地治理专项资金1500万元，增长40％。“集体经济百强村”创建活动深入开展。“千村扶贫”工程有效实施，帮助全市40个贫困村发展经济。

商贸服务业持续发展。商业资源优化整合，有效拉动消费，社会消费品零售总额达到254．5亿元，增长5．8％。克服非典影响，下半年会展业持续兴旺，纺织服装、玩具、塑料机械、东盟商品、美食等展会相继举行。旅游业稳步发展，接待旅游者248．2万人次，旅游收入37．8亿元。我市被授予“中国潮菜之乡”称号。交通运输业进一步发展，货运量1463．8万吨，增长2％；港口货物吞吐量1468万吨，增长6．4％，其中集装箱22．3万标箱，增长8％。通信产业层次不断提升，电信服务业保持增长。建筑业向外拓展势头强劲，外地施工产值61亿元，增长14％。

———招商引资掀起新高潮

招商引资取得成效。招商引资呈现外资侨资多、民资内资多、增资扩产项目多和大项目多的新气象。举办“6·28”和“10·18”两次大型招商和项目推介活动，取得了良好效益。金威啤酒等一批项目落户汕头。合同利用外资9．9亿美元，出现大幅度增长；实际利用外资2亿美元，增长27．6％。新引进外商直接投资项目64个，其中1000万美元以上项目12个。金融支持力度加大，银行与企业合作有新进展。国家开发银行为我市提供五年贷款额度80亿元。对外贸易快速增长。积极落实扩大出口扶持政策，重点抓好年出口300万美元以上企业在退税、通关、金融等方面的服务协调工作。外贸进出口总额33．5亿美元，增长15．8％，其中出口总额18．9亿美元，增长20．6％。私营企业出口保持高速增长，占出口总额的28．7％，成为外贸出口新亮点。机电产品出口活跃，出口市场、产品结构不断优化。

国际交流合作进一步加强。坚持借侨兴市，积极组团参加第十二届国际潮团联谊年会。加强与港澳台的经济文化交流合作，出访韩国、日本、新加坡、马来西亚、泰国等取得圆满成功，汕头对外影响力日益扩大。

———城市建设和生态环境建设取得新成效

行政区划调整顺利完成。实施行政区划调整，各区机构设置、人员定岗分流、理顺管理关系等工作有序进行。市区面积从289．9平方公里扩大到1956．4平方公里，城市发展空间进一步拓展，为优化资源配置和产业布局提供了有利条件。

城市化步伐加快。着力构建以金平、龙湖、濠江为城市中心，澄海、潮阳、潮南城区为副中心，南澳为前沿，中心镇和一批新兴城镇为骨干的市域城镇体系。落实扶持中心镇发展的政策措施，中心城镇建设有新进展。乡镇撤并工作顺利进行，农村城镇化进程加快。

基础设施建设积极推进。实施投资拉动，全社会固定资产投资119亿元，增长10．2％。加强交通能源水利设施建设，总投资171亿元的54项重点项目建设进展顺利。汕头港总体规划获交通部批准，汕头干线公路网修编基本完成。广澳港区一期工程、汕头港主航道及外拦沙堤、珠池港区二期工程等重点项目建设取得较大进展。广澳港5万吨级主航道工程等项目前期工作正在抓紧进行。华能汕头电厂二期工程动工建设。汕头大围等一批水利建设项目前期工作进展顺利。外砂桥闸重建等水利工程项目建成投入使用，一批江海堤围、小型水库完成除险加固工作。

市政设施建设取得成效。完成解放桥维修加固和黄河路东延工程，推进金凤路桥、华山北路和续建黄山路、汕樟北路等市政工程项目。石大桥北引桥动工建设。市客运中心站等枢纽站场建成运营。旧城区改造步伐加快，改造竣工新楼房建筑面积56．3万平方米。 环境和资源保护工作进一步加强。开展水污染专项整治，加快整治练江、濠江、潮阳护城河、澄海环城河的前期工作；中心城区新河沟、龙湖沟等江河整治力度逐步加大。环保基础设施建设步伐加快，雷打石生活垃圾处理场首期工程建成投入使用，市特种废弃物处理中心动工建设，澄海、潮南、南澳等一批环保设施工程建设进展顺利。城市环境综合整治定量考核成绩居全国前列。完成一批水源涵养林、沿海红树林工程。南澳县创建国家生态示范区和国家4A级旅游区工作通过省级初审，启动设立西环山、大南山市级森林公园工作。加强耕地保护，实现占补平衡。

———改革发展迈出新步伐

民营经济加快发展。出台《关于加快民营经济发展的决定》，配套扶持措施，促进民营企业发展。全市民营企业投资1000万元以上的项目84个，总投资额32．5亿元。列入省、市资金扶持的中小企业技术改造和技术创新项目60个，投资额17．9亿元。民营企业参与国有企业改革有新进展。民营工业产值占全市工业总产值的78．5％，民营经济不断壮大。

体制改革继续深化。国有企业改革稳妥推进，市直完成产权改革的公有制企业19户，已进入改革程序的公有制企业52户。积极探索运用资本运作推进国企改革的新路子。改革投融资体制，鼓励各类投资者参与城市基础设施和公共设施建设。扎实推进农村税费改革，合理确定税负，规范各种农村收费，农民减负率达84．2％。积极开展调整市、区管理权限和财政体制改革的调研工作。

市场经济秩序进一步规范。开展专项整治，个别地方存在的走私贩私、制假售假等经济违法犯罪活动得到有效遏制。积极稳妥地解决历史遗留问题，地方金融资产保全清收工作力度加大，市商业银行处置工作加紧进行。建设工程招投标管理制度进一步完善。工商、物价、统计、审计、监察工作更好地为经济社会服务。信用建设稳步推进，信用服务市场化运作开始起步。

———精神文明建设和民主法制建设结出新硕果

思想道德建设不断加强。全市兴起学习贯彻“三个代表”重要思想新高潮，公民道德建设活动广泛开展。“汕头人精神”大讨论引起强烈反响。群众性精神文明创建活动广泛开展，龙湖区等一批文明单位受到省委、省政府表彰。军民共建活动成效显著，第三次蝉联“全国双拥模范城”称号。

民主建设继续推进。坚持向人大及其常委会报告工作、向人民政协通报情况，自觉接受监督。加强与工会、共青团和妇联等群众团体的联系，广泛听取各方面对政府工作的意见。人民代表议案（建议）82件、政协委员提案204件全部办理完毕。基层民主继续扩大，村民自治、社区自治组织建设得到加强。澄海区被授予“全国村民自治模范区”称号。

法制建设步伐加快。提请市人大常委会审议法规草案2件，颁布政府规章8件、规范性文件28件。重大决策法律审查和规范性文件审查工作全面铺开。规范行政执法行为，强化行政执法监督，落实行政执法责任制与评议考核制，行政复议工作更加规范，政府依法行政水平得到提高。实施第三轮行政审批制度改革，削减审批事项176项。城市管理相对集中行政处罚权试点工作不断推进。普法教育和法律服务工作扎实开展，全民法律意识普遍提高。

政府作风有新转变。县级以上政权机关全面推行政务公开。改进机关作风、优化投资环境专项活动取得成效。廉政建设和反腐败斗争深入开展，廉政建设责任制进一步落实。查处违纪违法案件，从政行为进一步规范。重视人民群众来信来访，积极稳妥化解人民内部矛盾，一批群众关心的社会热点问题得到解决或正在解决。

社会治安综合治理进一步加强。治安防控体系建设扎实推进，社会治安综合治理责任制有效落实。保持高压态势，严厉打击暴力犯罪和“两抢”、盗窃机动车等各类刑事犯罪活动，社会治安秩序好转。

———社会各项事业取得新进展

科技创新成果显著。大力实施技术创新工程，高新技术和先进适用技术改造提升传统产业步伐加快。分别新增国家级、省级高新技术企业2家和14家。新增科技成果73项，获省科学技术奖6项，推广转化科技成果106项。组织实施各类科技发展计划，其中列入国家级科技计划项目27项、省级75项。全年专利授权量1962件。我市被确定为全省唯一的知识产权试点城市。

教育事业不断推进。基础教育管理体制进一步完善，中小学布局调整优化。素质教育得到加强，常规教育在全省保持优势地位，高考成绩名列全省前茅。教育结构逐步优化，职业教育和成人教育巩固发展。教育综合改造工程和信息化建设取得新进展。投入校舍建设资金2．9亿元，竣工面积32．8万平方米。中小学在校生增加80832人，入学难问题逐步缓解。汕头大学办学水平进一步提高。

文化事业和文化产业稳步发展。群众性文化活动蓬勃开展，成功举办第二届国际民间音乐花会和国际灯谜节。文化艺术、新闻出版、广播电视、社会科学等在经济社会发展中发挥积极作用。文化管理体制改革逐步推进，基层文化建设落后状况有所改善。文化产业初具规模。“扫黄”、“打非”工作取得实效，文化市场在规范中健康发展。

卫生、体育等各项事业发展步伐加快。非典疫情有效控制。制定全市防治非典应急预案，公共卫生体系建设不断加强。城镇医疗卫生体制改革逐步深化，农村卫生设施建设扎实推进，三级医疗预防保健网络不断完善。计划生育工作得到加强，人口出生率11．7‰，人口自然增长率6．8‰，完成省下达的任务。殡葬改革取得明显成效。国际渡海节等群众性体育活动广泛开展。成功承办全国青少年花样游泳比赛和少儿跳水锦标赛。竞技体育水平不断提高，我市运动员共夺得世界冠军3个、亚洲冠军4个、全国冠军25个。人民武装工作积极推进，顺利完成征兵工作，民兵预备役工作进一步加强。民政、人民防空、妇女儿童、残疾人事业、宗教、气象、地震、保密、档案、地方志等工作取得新成绩。

人民生活有所提高。社会保障体系进一步健全，落实就业再就业扶持政策措施。全市城镇新增就业6．6万人，城镇登记失业率控制在3．2％以内。征收社保费7．9亿元，增长21．6％；企业离退休人员养老金按时足额社会化发放；实施低保48667人，增长14．6％。年初确定的8个为民办好事实事项目基本完成，进一步缓解了城乡居民住房难、饮水难等热点问题。解决和落实市级以上劳模待遇。农民和城镇居民生活水平有所提高，中心城区居民人均可支配收入9105元，农村居民人均纯收入4064元，分别实际增长4．4％和4．6％。

各位代表！过去的一年，我们取得了可喜的成绩，这是省委、省政府和市委正确领导的结果，是市人大及其常委会和人民政协支持监督的结果，是全市人民同心同德、艰苦奋斗的结果，是海内外各界支持和帮助的结果。在此，我代表市人民政府，向给予政府工作大力支持的人大代表和政协委员，向全市广大工人、农民、知识分子、干部、驻汕人民解放军、武警官兵、公安干警，向中央和省驻汕单位、各民主党派、工商联、人民团体以及社会各界人士，表示崇高的敬意！向关心和支持汕头建设的港澳台同胞、海外侨胞和国际友人，表示衷心的感谢！

我们也必须清醒地看到，我市经济社会发展还存在许多困难和问题。主要是：经济总量不大，综合竞争力不强，经济增长速度低于全国、全省平均水平；产业基础尤其是工业基础比较薄弱，产品附加值低，经济效益不高；人口基数大，就业压力大，城乡居民增收幅度小；财政仍较困难，市商业银行的处置任务艰巨；经济和社会管理的任务较为繁重，维护稳定的工作仍需进一步加强；营商成本相对较高，投资软环境仍有待优化。对此，我们必须高度重视，切实认真加以解决。

2004年工作安排

2004年，是我市继续深入学习贯彻十六大和十六届三中全会精神，加快汕头经济社会发展的关键一年。

新的一年，汕头加快发展将迎来新的机遇。十六届三中全会通过的《关于完善社会主义市场经济体制若干问题的决定》，必将为汕头加快发展注入新的动力。内地与香港、澳门关于建立更紧密经贸关系安排的实施，我省加快推进东西两翼发展，为汕头加快发展提供更加广阔的空间。当前，全市经济加速增长态势明显，人心齐、干劲足。汕头正蓄势待发，处在大发展的前夜。同时，我市经济发展也面临着压力和挑战：世界贸易摩擦进一步加剧，对我市的纺织服装业、水产品加工业等将产生直接冲击；国家调整外贸出口退税政策，将给我市扩大出口带来影响；周边城市发展势头迅猛，地区竞争压力不断增大。应该看到，加快推进汕头经济社会发展任重道远，必须经过长期坚韧不拔的艰苦努力。因此，我们一定要进一步解放思想，与时俱进，树立强烈的发展意识、特区意识和危机意识，以饱满的热情和创新的精神谋一域之发展，在新的起跑线上实现新跨越。

2004年政府工作的总体要求是：以邓小平理论和“三个代表”重要思想为指导，全面贯彻中共十六大、十六届三中全会和省委九届四次全会、市委八届二次全会精神，坚持以人为本，牢固树立和落实全面、协调、可持续的科学发展观，保护好、引导好、发挥好各方面加快发展的积极性，深化改革，扩大开放，调整优化经济结构，大力发展临港工业，逐步建立战略产业，促进现代产业协调发展、城乡经济整体推进，提高人民生活水平，推进政治文明和精神文明建设，实现国民经济持续协调健康发展和社会全面进步。

根据上述总体要求，围绕加快建设经济强市和区域性中心城市的目标，必须大力促进经济工作“四个转变”：经济结构从单一经济、小工业为主向多元经济、大工业和支柱产业转变，构建汕头经济战略产业；项目布局从分散向园区聚集转变，真正把工业园区建设成为推动汕头经济发展的引擎和加速器；招商引资从局部区域为主向民资侨资、内资外资并举转变，增强投资原动力；经济增长从数量扩张向数量质量并重转变，提高经济发展的质量和效益。努力实现“四个协调”：城乡协调发展，区域协调发展，经济与社会协调发展，人与自然协调发展。

今年全市经济和社会发展的主要预期目标是：全市生产总值增长11％，其中第一产业增加值增长3％，第二产业增长13％，第三产业增长10％；地方一般预算收入增长11％；社会消费品零售总额增长11％；外贸出口增长10％；全社会固定资产投资增长12％；新增城镇就业岗位6．5万个；城镇登记失业率控制在3．5％以下；人口自然增长率控制在8．3‰以下；城镇居民人均可支配收入增长6％，农村居民人均纯收入增长3％。

为实现上述目标，必须抓好以下工作：

一、全面实施工业强市战略，推动工业上规模上水平

以园区经济推动产业集群发展。大手笔、大气魄整合园区资源，调整园区布局，高起点、高标准规划建设高新技术和先进工业园区。重点抓好濠江石化园区、澄海岭海工业园区和潮阳、潮南工业经济带的规划建设。要以园区吸引项目，以项目支撑园区发展。突出园区产业特色，培育产业生态，加强各园区间上下游产业的衔接，延长产业链，推动产业集聚和中小企业集群发展。完善园区环境，建设生态型工业园区。下大力气办好保税区、高新区。

以结构调整促进产业优化。调整优化产业结构，构建具有汕头特色、比较优势和较强竞争力的新型工业体系。大力发展临港工业、石化工业、海洋产业等事关汕头长远发展的战略产业，扶持发展制造业特别是装备制造业、信息制造业和船舶制造业。继续发展劳动密集型工业、轻型加工业，引进和发展高新技术产业和先进工业，改造、提升、壮大传统优势产业。做大做强支柱产业，培植一批有影响力的特色行业，推进纺织服装、工艺玩具、音像材料产业基地建设。重点加快金威啤酒、超声超扭曲液晶显示器等项目建设，加紧跟踪落实铝箔等一批重点工业项目。打破地区、行业、所有制界限，引导企业实现资源优化配置，重点扶持一批有发展潜力的大型企业集团。

以品牌带动形成产业优势。鼓励企业广泛应用信息技术、高新技术和先进适用技术，提高核心技术和重要应用技术的创新能力，培育名牌和拳头产品。大力开发应用专利技术，扶持一批带动能力强、拥有自主知识产权的重点行业、重点企业和重点产品。引导宜华等一批企业实施以科技为内在支撑的名牌战略。加强对名牌产品保护，重奖创立名牌产品企业，争创更多国家级、省级品牌。

二、深化体制改革，增强经济发展活力

加快国有企业改革。以产权制度改革为突破口，推进产权多元化，努力使股份制成为公有制的主要实现形式。盘活国有资产，优化股权结构，实现国有资产合理配置及保值增值。采取资产重组、资本运作、产权转让等方式，拓宽国有资本退出通道。鼓励和引导企业员工、经营者出资参股，吸引社会资本参与国企改制。规范国企改制程序，完善国有资产评估机制，防止国有资产流失。加快建立健全国有资产管理体制，实现管人、管事、管资产相结合。成立市国有资产监督管理机构，履行国有资产出资人职责。完善公司法人治理结构，取消国有企业行政级别，实行分类定级。妥善处理改制企业的债权债务和职工社会保障、再就业、经济补偿等问题。

大力发展民营经济。进一步落实加快民营经济发展的政策措施，放宽市场准入，加强服务，努力为民营企业创造良好的发展环境。完善民营企业管理服务体制，重点扶持科技型、外向型、就业型、农产品加工型民营企业发展。坚持扶优扶强和联合发展，鼓励优势民营企业收购、兼并国有、集体企业，培植一批跨地区、跨行业、跨所有制、具有核心竞争优势的民营企业集团。继续引导民营企业规范运作，守法经营。推进民营企业技术创新，增强市场竞争力。

创新投融资机制。放开投资领域，拓宽投融资渠道，培育社会投资主体。积极利用银行贷款、企业债券、BOT、TOT等投融资方式筹措资金。盘活政府性资源，选择一批市政、环保、公路、港口等建设项目向社会公开推介，鼓励社会资本以独资、合资、合作、联营、特许经营、项目融资等方式参与建设和经营。精心组织好项目的推介和招商工作，争取更多的项目纳入国家、省计划盘子。

调整市、区管理权限。尽快出台调整市、区两级部分管理权限的意见，理顺市、区两级的财权、事权、职能、职责，进一步简政放权。市一级主要集中抓好规划、调控、监管工作和重大基础设施建设，充分调动市、区两级以及各方面的积极性，提高办事效率。

三、加强规划建设，提高城市化水平

规划建设三条经济带，形成大汕头发展格局。根据行政区划调整后汕头发展的新格局，加快编制全市概念性规划和近期建设规划，推动组团式梯级发展，提高城市化水平。着力规划建设三条经济带，即海湾大桥至澄海莱芜的城市经济带，建成高标准的防洪大堤、高等级的市政大道和亮丽的生态海滨景观长廊，促进中心城区东扩；深汕高速公路潮阳、潮南路段以北的工业经济带，建成工业经济走廊，成为潮阳、潮南发展和招商引资的桥头堡；南滨路西延穿越濠江、潮阳、潮南的经济带，建成城市组团、高新技术产业组团。要把建设三条经济带作为一项长远规划，实施东延、西连、南拓、北优，全方位打造大汕头城市景观，促进东西两域协调发展。

强化经营城市理念，提高城市管理水平。全面整合城市资源，实行土地资源、海洋资源、城市公共资源、旅游资源等政府可支配资源的有偿使用。创新城市管理体制，推进城市资源市场化运营。创新政府管理投资项目的方式，鼓励引导社会资本参与城市基础设施建设。推进中心城区环保基础设施等重点城市建设工程。动工兴建兰水星主题公园。着力抓好海滨路西延和小公园历史建筑风貌保护区改造工程，加快旧城改造步伐。解决好“城中村”问题。认真解决中心城区“断头路”和路灯照明问题。加大城市绿化美化力度，在中心城区逐步推行“一路一树种、一路一灯饰、一路一景点”，致力打造独特的城市形象品牌。

实施投资拉动，全力推进基础设施建设。适度超前规划建设一批重大基础设施和市政设施。加快交通设施建设，完善海陆空一体化交通网络。加快广澳深水航道的疏浚和5万吨级码头的建设，10万吨级泊位和30万吨级油码头的规划论证。争取今年3月开通汕头—香港海上邮轮，积极争取空港增加航线、航班，形成海港、空港“两港齐飞”的格局。加快金凤—西港路桥、金鸿公路、海滨路拓宽和延伸等工程建设，扩建国道324线外砂等桥梁；抓紧规划建设汕揭梅高速公路汕头段，协调做好汕头—深圳、汕头—漳州等沿海铁路的前期规划。推进能源电力建设，重点加快华能汕头电厂二期工程进度，做好华能汕头第二电厂前期工作，加快南澳风能发电项目建设。加强水务工作，推进城乡水利防灾减灾工程建设。加快一批江河海堤的规划整治，重点抓好牛田洋海堤、汕头大围的建设。加快实施省人大小型水库除险加固和机电排灌改造议案。加快乌桥岛堤围建设。继续抓好韩江下游出海口东里等桥闸的重建改造工程。抓紧潮阳、潮南引韩供水工程的前期工作，做好南澳引韩跨海工程的规划、论证。加快续建第二过海水管的前期工作，加速向濠江、潮阳、潮南供水。抓好新一代多普勒雷达系统建设，增强防灾减灾能力。

四、高度重视“三农”问题，推进农村城镇化和农业产业化

壮大区县域经济，统筹城乡发展。以行政区划调整为契机，根据各区县的区位和资源优势，科学规划，准确定位，合理布局，优化产业，培育各具特色的区县域经济。加强各区县间产业优势互补和辐射联动，提升汕头整体发展水平。强化城乡规划，优化城镇布局和资源配置，加大城镇基础设施建设力度，形成科学合理的城镇体系。打破城乡二元结构，消除城镇化进程中的制度性障碍，推进城乡一体化。加快发展一批中心镇和专业镇，继续抓好并镇并村工作。发展劳务经济，促使农业人口向城镇转移、农村劳动力向工业和园区转移，实现城市工业集群化与农村工业化互动，城市服务业发展与农村劳动力转移互动，城市组团发展与农村城镇化互动，推进城乡协调发展。

发展特色农业，大力推进产业化经营。继续调整优化农业产业结构，培育和发展蔬菜、水果、畜牧、水产四大支柱产业，拓展“品牌农业”。实施优势农业区域化发展规划，建设高标准农田和一批名优农产品基地。努力推进农业标准化生产。构建农产品加工体系，培育以市场、加工、科技带动型为主的农业龙头企业。引导社会资本创办农产品加工业和农村服务业，形成以优势农产品为依托、市场占有率高的食品加工业。大力发展海洋渔业，争取海门渔港列入国家级中心渔港规划，做好云澳渔港列入国家级中心渔港的前期工作。加快农村基础建设，完善农村社会化服务体系。

切实减轻农民负担，增加农民收入。坚持“多予、少取、放活”的方针，逐步建立农民增收的长效机制。深化农村税费改革，落实农民减负领导责任制和部门专项治理责任制。继续清理整顿涉农行政性和经营服务性项目收费，完善涉农收费公示制。继续扶持113个集体经济年纯收入3万元以下的贫困村尽快脱贫。加强教育、科技、文化扶贫，推进产业化扶贫。拓宽农民就业门路，增加农民非农收入。

五、进一步扩大开放，千方百计招商引资

优化进出口结构，拓展国际市场。深化外贸体制改革，推进外贸经营主体多元化。全面实行特区进出口经营权核准登记制，扩大自营进出口的民营企业群体。优化出口市场结构，在巩固东南亚、北美和欧盟等重点市场的基础上，加大对俄罗斯、非洲、中东及南美等市场的开拓力度。优化出口产品结构，增加高新技术产品出口。促进加工贸易转型升级。大力发展一般贸易和服务贸易，支持企业开展对外劳务输出和工程承包。发挥口岸优势，千方百计做大进口。

加强对外经贸合作，做好“两个承接”。发挥侨乡优势，通过走出去、请进来，大力吸引投资。以CEPA实施为契机，大力提升汕港澳经贸合作水平。加强对台经济技术交流合作。抓住建立东盟自由贸易区的契机，加强与东南亚的经济合作。主动承接欧美、日韩、东南亚、港澳台的产业转移，承接珠三角地区的产业转移。

优化招商环境，提高利用外资的质量和水平。坚持内资和外资并举、规模和质量并举、引资和引智并举，广泛吸引社会资本投资兴业。开辟招商新途径，通过园区招商、产业链招商、委托代理招商、网上招商等方式，争取在引进和发展战略性产业上有新突破。要以乡情促商情，把侨乡优势转化为推动汕头发展的强大动力。强化服务，鼓励现有企业增资扩产。推进“大通关”建设，改革通关模式。建立激励机制，落实招商责任，形成全社会招商引资的合力。

六、加快发展服务业，提高第三产业整体水平

大力发展服务业。规划论证汕头大会展业，发展会展经济。继续办好汽车、食品、毛衫服装、玩具礼品等大型展会。整合旅游资源，建设“中国美食之都”。大力发展具有潮汕文化特色的滨海旅游，办好第二届汕头旅游节。抓好南澳4A级旅游区建设。发展现代物流业，建设现代物流园区。优化港口资源，积极拓展远洋近洋运输。培育发展各类社会中介组织。加快信息业等新兴行业的发展。完善土地、技术、劳动力、人才等要素市场。健全产权交易市场，规范产权交易行为。

着力提升传统商贸业。深化流通体制改革，引导国有资本有序退出商贸业。整合优化商业资源，扶持发展一批商贸龙头企业。大力培育和发展大型商贸市场以及玩具礼品、纺织服装等专业市场。创新商贸方式，大力推进连锁经营、电子商务等现代营销和流通方式，发展现代流通业。

努力开拓国内市场。积极参与区域合作，加强与国内各地的经济交流，推进与闽粤赣边地区的经济协作。通过举办经贸洽谈会和商品博览会，不断提高汕货的影响力和市场占有率。引进大型消费网点，繁荣本地市场。扩大农村消费，拓宽消费品进入农村市场渠道。大力培育住房、汽车等消费信贷，催生消费新增长点，扩大消费需求。加强商品质量监督，维护消费者合法权益。大力开拓国内建筑市场，打响汕头建筑品牌。

七、强化财税金融工作，为加快发展提供财力和金融支持

规范税收征管。千方百计拓宽税基，涵养税源，优化税收结构，确保应收尽收，提高财政一般预算收入占全市生产总值的比重。完善税收属地征管，严格依法治税，强化重点税源监控，加大征收稽查检查力度。大力清缴欠税，堵塞征管漏洞，做到依率计征。

加强财政管理。坚持依法理财，调整优化财政支出结构。严格预算管理，确保重点支出资金需要。增收节支，堵塞跑、冒、滴、漏，用好纳税人每一分钱。卫生、教育、文化等公共事业性经费增加部分重点向农村倾斜。推进“收支两条线”管理改革，动真格清理和规范行政事业性收费。开展财政支出的效益审计，严肃财经法纪，加强对国债项目、专项资金的管理，加大对财政性投资工程建设项目的监督力度。

推进公共财政体制改革。建立以国库单一账户体系为基础的财政国库管理制度，提高财政性资金的运行效率和使用效益。推行部门预算改革，加大预算内外资金综合统筹管理力度，继续扩大政府采购范围和规模。采取积极财政措施，利用财政杠杆，激活社会资本。

加大金融的支持力度。进一步完善政府、银行和企业协调制度，促进金融资产良性循环，提高信贷资金使用效益。加强与金融机构的合作，抓紧落实国家开发银行与我市合作协议项目。积极探索建立市场化的中小企业信贷担保机构，解决企业融资难问题。继续防范和化解地方金融风险，严厉打击逃废金融债务不法行为。积极推进汕头商业银行的最终处置和汕头国投的重组工作。

八、以人为本，推进社会各项事业

加强公民思想道德建设。深入开展“三个代表”重要思想学习教育活动，推进多形式的精神文明创建活动。继续实施《公民道德建设实施纲要》，弘扬“汕头人精神”，提升全民的道德素质和精神境界。普及科学知识，倡导科学、文明的生活方式，提高公民现代文明素质和城乡文明程度。

加快文化大市建设。围绕建设文化大市目标，抓紧出台《关于加快建设文化大市的决定》。深化文化体制改革，建立与市场经济相适应的文化管理体制。实施精品战略，繁荣文艺创作。坚持继承和创新相结合，开展“潮学”研究，弘扬优秀潮汕文化，不断提高潮汕文化的品位，推动传统文化与现代经济的有机融合。发展群众文化，丰富群众文化生活。培育壮大文化产业，对经营性文化行业逐步实行企业化、市场化、社会化改革。大力开发文化产品，扩大文化需求。强化文化市场管理。加大文化设施建设力度，重点抓好市图书馆、博物馆等工程的配套建设。推动对外文化交流。

推动科技创新。继续深化科技体制改革。加大对企业技术创新的引导和扶持力度，积极开展企业技术创新试点工作，加强产学研合作，完善科技创新体系。继续扶持科技园区和软件园建设，增强其孵化作用和技术支撑作用。大力推进国民经济和社会信息化建设。建立科技成果转化服务平台，促进科技成果转化为现实生产力。重视知识产权工作，加大力度保护知识产权。

加快教育改革和发展。推进教育改革创新，全面实施素质教育。大力发展农村教育，完善“以县为主”的基础教育管理体制。巩固提高“普九”成果，大力发展高中阶段教育。优化配置教育资源，改善教育环境和办学条件。建设高素质师资队伍，提高教育质量。加快教育信息化步伐。调整中小学布局，抓好全市新建、扩建、改建、续建的40所重点校舍建设项目。各级财政要加大对基础教育和农村教育的投入，解决好“入学难”和“大班额”问题。加快潮阳镇二小学新校舍建设。加大农村“并校”工作力度。推动社会力量办学，完善多元化、多渠道投资办学的格局。发展职业教育和成人教育，构筑终身教育体系。办好汕头职业技术学院。继续支持办好汕头大学，发挥其人才库和科研基地的作用。

大力实施人才战略。确立人才资源是第一资源的观念。深化人才管理制度改革，尽最大努力培养、吸引、使用和留住人才。以行政管理人才、企业经营管理人才和专业技术人才为主体，建设规模宏大、结构合理、素质较高的人才队伍。完善激励机制，实行公平竞争，积极为各类人才营造良好的政策环境、工作环境、人际环境、生活环境和社会环境。建立人才创业服务体系，提供人才创业的良好平台。

加快发展卫生体育等各项事业。加强疾病预防控制体系、卫生执法监督体系和医疗救治体系建设，完善公共卫生事件应急处理机制，提高应对各类突发公共卫生事件的能力。继续做好防治非典工作。启动高致病性禽流感防治应急预案，落实各项防治措施。完成市疾控中心易址重建工程和中心医院传染科的改建工程。健全城市基本医疗服务体系，加快新型农村合作医疗制度建设步伐。增加群众性体育设施，发展体育产业。办好2004年全球华人篮球邀请赛。加快中国乒乓球汕头训练基地建设。做好体育公园规划和征地等前期工作。加强国防动员和全民国防教育工作，抓好民兵、预备役建设。巩固双拥工作成果，做好拥军优属、优抚安置工作。重视和做好宗教、对台、人防、外事、侨务、统计、物价、档案、保密、地方志等工作。

九、关注民生，努力提高城乡群众生活水平

努力扩大就业和再就业。认真落实促进就业再就业各项优惠政策，努力改善就业环境。推行灵活多样的就业形式，拓宽就业渠道，扩大就业容量。完善就业服务体系，健全街道（镇）劳动保障机构。完善再就业小额担保贷款制度，鼓励、扶持自主创业。加强再就业培训和职业技能培训。完善市场导向的就业机制，积极推进城乡统筹就业。

继续完善社会保障体系建设。巩固和扩大社会保险覆盖面，完善养老、失业、工伤、生育和医疗保险制度。加强社会保险费征管，实现全市社会保险市级统筹。确保国有企业下岗职工基本生活，确保离退休人员养老金按时足额发放，确保符合条件的参保人员的待遇应发尽发、应保尽保。推进企业退休人员社会化管理服务。规范和完善城乡居民最低生活保障制度，将所有符合条件的贫困群众纳入低保范围。

解决好群众关注的热点难点问题。按照整体规划、统一部署、分步实施的原则，积极实施全民安居、扩大与促进就业、农民减负增收、教育扶贫、济困助残、外来员工合法权益保护、全民安康、治污保洁、农村饮水、城乡防灾减灾等“十项民心工程”。关心群众生活，切实解决饮水难、入学难、住房难、就医难、行路难等问题。落实归还华侨业主侨房使用权。大力实施“星光计划”，重视做好老龄工作。

十、坚持可持续发展，促进人与自然的和谐

加强环境保护和治理。强化城市环境综合治理，加大对工业污染源和大气、水、噪声污染整治力度。加强建设项目的环保管理，从源头上控制新污染源。推进环保基础设施建设，加快雷打石垃圾处理场二期工程和澄海、潮阳、潮南、南澳污水处理厂建设；抓紧建设西区污水处理厂、水鸡岭垃圾处理场和澄海、潮南、南澳垃圾处理场。理顺雨污分流排放体系，加大对三脚关沟、新河沟、龙湖沟、港区排洪沟的整治力度。保护韩江流域水质，整治练江、濠江。加强城乡环境卫生管理。

加快生态建设步伐。全面实施“青山碧水绿化美化”工程，加大保护水资源和自然生态环境力度，使汕头天更蓝、水更清、地更绿、城更美。加强城乡造林绿化，大力实施生态公益林体系建设规划。推行水源、海域等不可再生资源的有偿开发和保护性利用，促进海洋资源开发和运用的良性循环。重视湿地、红树林资源保护，加快自然保护区和森林公园建设。继续推广生态示范区的创建经验，抓好南澳“全国生态示范区”的创建工作。积极创建国家园林城市、国家生态城市。建立和完善自然灾害监测预警机制。继续推进殡葬改革。

切实加强土地管理。加强土地利用总体规划管理，从严控制用地规模，实施土地用途管制。合理配置国土资源，加大力度处置闲置土地，促进土地的合理、高效和可持续利用，为加快工业化、城镇化进程提供用地保障。落实耕地保护制度，加大整理复垦补充耕地力度，确保耕地总量动态平衡和占补平衡。大力整顿和规范土地市场秩序，加强土地市场宏观调控力度。严格实行经营性用地招标、拍卖、挂牌出让制度，对全市土地市场实行集中统一管理。严格依法征地，积极稳妥推动集体土地使用权流转，保护农民合法权益。

加大人口和计划生育管理力度。倡导优生优育，提高人口素质，严格控制人口增长。落实各级人口与计划生育目标管理责任制，坚决执行“一票否决制”，确保完成年度计生工作任务。加强计划生育服务中心和基层计生站、所建设，加强计划生育队伍建设，落实基层计生专干报酬，推动计划生育工作重心下移。坚持经常性工作和集中服务活动相结合，重点加强计划生育薄弱环节。对计生重点地区，要分片包干、挂点包干，尽快扭转计生工作被动局面。加大计生投入，落实计生专项经费。加强对流动人口的计划生育管理。力争经过3年的艰苦努力，使我市计生工作达到全省中上水平。

十一、依法行政，推进民主法制建设

加强民主制度建设。自觉接受人大及其常委会的监督，认真执行人大及其常委会的决议、决定，及时向人大及其常委会报告工作。积极支持人民政协履行政治协商、民主监督、参政议政职能，主动听取各民主党派、工商联、无党派人士和各人民团体的意见建议。办好人大代表议案、建议和政协委员提案。逐步建立重大事项社会公示制度和社会听证制度，扩大公民有序的政治参与。坚持政务公开，完善政府决策咨询机制和重大经济社会问题的科学化、民主化、规范化决策程序。继续加强基层民主建设，推进乡镇政务公开、村务公开，特别是农村财务、征用土地等直接涉及农民利益事务的公开。健全职工代表大会和其他形式的企事业单位民主管理制度。

严格依法行政。认真贯彻实施行政许可法，清理、修改、废止与行政许可法不相一致的规定，清理行政许可实施机构。创新行政许可的体制和机制，推行统一办理、联合办理、集中办理行政许可。加强政府法制工作和执法监督，规范行政复议和行政应诉，提高依法行政水平。继续深化行政审批制度改革。推进相对集中行政处罚权和行政执法评议考核工作。

推进政风建设和廉政建设。牢记“两个务必”，坚持为民、务实、清廉，自觉为市民服务、为企业服务、为纳税人服务。加快转变政府职能，提高政府驾驭市场经济和应对复杂局面的能力，提高社会管理和公共服务水平。改进机关作风，提高办事效率，确保政令畅通。厉行节约，严禁奢侈浪费。加强廉政建设，落实廉政责任制和廉洁自律的各项规定。强化监察和审计工作，加大对违纪违法案件的查处力度。坚决纠正损害人民群众利益的不正之风。

强化社会管理。依法规范市场经济秩序，继续在重点地区、重点行业开展打击制假售假、走私贩私等违法犯罪活动。整治垄断、欺诈等不正当竞争行为。推进社会信用体系建设，全面提升企业、组织和个人的公信力。构建完善的市场监管体制，切实保护投资者、经营者和劳动者的合法权益。落实安全生产责任制，巩固安全生产工作成果。切实做好维护社会稳定工作，把妥善处理人民内部矛盾摆在突出位置，把影响稳定的因素解决在基层、化解在萌芽状态。落实社会治安综合治理责任制，坚决打击各种刑事犯罪活动，重点打击“两抢”和社会黑恶势力。继续打击“六合彩”赌博活动。加大对治安突出问题和重点区域的整治力度，完善社会治安防控体系，建立健全群防群治网络，及时消除治安隐患。

各位代表！振兴经济，加快发展，是480万汕头人民的迫切愿望，是历史赋予我们的神圣使命。我们要十分珍惜今天来之不易的良好局面，更加紧密地团结在以胡锦涛为总书记的党中央周围，在邓小平理论和“三个代表”重要思想指引下，在省委、省政府和市委的正确领导下，振奋精神，凝心聚力，励精图治，众志成城，在新的起跑线上努力开创汕头现代化建设的新局面！
